# Experimental study of cBMMSC based on nanosilver hydrogel nerve conduit for repairing spinal cord injury

**DOI:** 10.1111/jcmm.70149

**Published:** 2024-11-26

**Authors:** Zhuxiao Tang, Yahui Ye, Kaichuang Yang, Xi Guo, Xin Gao, Cheng Wu, Jingyu Wang, Deqing Peng

**Affiliations:** ^1^ Brain Center Zhejiang Hospital Hangzhou Zhejiang China; ^2^ School of Public Health, Hangzhou Medical College Hangzhou Zhejiang China; ^3^ Center for Rehabilitation Medicine, Department of Neurosurgery Zhejiang Provincial People's Hospital (Affiliated People's Hospital, Hangzhou Medical College) Hangzhou Zhejiang China; ^4^ Department of Neurosurgery The 2nd Affiliated Hospital of Zhejiang University Hangzhou Zhejiang China

**Keywords:** cBMMSC, nanosilver hydrogel, nerve conduit, spinal cord injury

## Abstract

Investigating the role of cranial bone marrow mesenchymal stem cells (cBMMSC) based on nanosilver hydrogel nerve conduits in the repair of spinal cord injury. Thirty adult Wistar rats, male and female, with body mass of 210‐240 g, were selected as experimental animals and divided into control group and experimental group, 15 rats each, by random number table method. The experimental group was treated with spinal cord injury and localized transplantation of cBMMSC‐containing nanosilver hydrogel nerve conduits, while the control group was treated with spinal cord injury and localized transplantation of cBMMSC‐free nanosilver hydrogel nerve conduits. Four weeks after transplantation, the expression of neuron‐specific enolase (NSE) in rat anti‐human nuclear monoclonal antibody (MAB1281)‐positive cells was detected by immunostaining in spinal cord tissue sections of the two groups to assess the differentiation of cBMMSC to neuron‐like cells in the nerve conduits after transplantation; the number of BrdU‐positive cells was detected to assess the neuronal regeneration of the localized spinal cord injury of the two groups; and the length of axons was observed with laser Confocal photography was used to observe the length of axons; HE staining was used to observe the scarring and cavities in the spinal cord sections of the two groups; immunofluorescence was used to detect neuron‐like markers (Nestin, NSE, NF200, GFAP) in the two groups, and the OD values were determined by the Image Processing and Analysis System (IPAAS); and Western blot was used to detect the neurotransmitters of motor fibres, acetylcholinesterase (ChAT), sensory fibres, and sensory fibres, as well as the neurotransmission of motor fibres, acetylcholine production‐limiting enzyme (ChAT) and sensory fibre neurotransmitter glutamate synthase (GOGAT); motor function was assessed by BBB score; somatosensory evoked potentials (SEPs) and motor evoked potentials (MEPs) were detected by body surface electrode assay in the two groups, and the neurophysiological recovery effect was evaluated. Four weeks after transplantation, the NSE content of MAB1281‐positive cells in the experimental group was significantly higher than that of the control group; the number of BrdU‐positive cells and axon length were significantly greater than that of the control group (*p* < 0.05); the scarring and cavitation of spinal cord slices were significantly lighter than that of the control group; the expression levels of Nestin, NSE, NF200, GFAP, ChAT, GOGAT, and BBB scores were significantly higher than that of the control group (*p* < 0.05); SEP and MEP latency time were significantly shorter than that of the control group (*p* < 0.05), and wave amplitude was significantly greater than that of the control group (*p* < 0.05). The cBMMSC transplantation based on nanosilver hydrogel nerve conduit was effective in repairing spinal cord injury, promoting neuronal and axonal regeneration, and restoring neuromotor and electrophysiological functions.

## 1 INTRODUCTION

The high incidence and disability rate of spinal cord injury has brought a heavy burden to patients' families and society, and its treatment and rehabilitation have now become a major difficulty in medical research. Clinically, the common methods of treating spinal cord injury include drugs, surgery and rehabilitation exercises, but they can only partially improve the symptoms, and it is difficult to recover the damaged spinal cord fundamentally. Most spinal cord injury patients still lose their neurological function, partly because the regenerative capacity of the spinal cord itself is very limited. Therefore, there is an urgent need for a novel and effective treatment for spinal cord injury to save the lost spinal cord neurological function.^1^, ^2^ In recent years, advances in stem cell transplantation technology have brought new hope for the treatment of patients with this disease. A variety of seed stem cells have been identified that can be used to repair spinal cord injuries, among which cranial bone marrow mesenchymal stem cell (cBMMSC) transplantation may be one of the attractive therapeutic options for repairing the injured spinal cord.^3^ cBMMSC have the advantages of easy access, easy transportation, low immunogenicity, no ethical controversy, non‐tumorigenicity, rapid proliferation and multidirectional differentiation. Animal studies have confirmed the great potential of cBMMSC to repair spinal cord injuries, but clinical translation has not been smooth, and the optimal pathway for transplantation of cBMMSC remains to be further explored.^4^ Studies over the past two decades have shown that axonal growth can occur after providing the correct substrate material following spinal cord injury. However, traditional tissue grafts or peripheral nerve grafts are often used to repair damage to the central nervous system, with a shortage of donors and frequent immune problems associated with infectious diseases.^5^ In recent years, nanosilver hydrogel nerve conduit, as a novel tissue engineering material, can be used as a medium for local cell transplantation with good histocompatibility, which provides new possibilities for the treatment of nerve injury.^6^ Based on the above background, in this study, cBMMSC were implanted into nanosilver hydrogel nerve conduits and transplanted into a rat model of spinal cord injury to investigate the repair effect on spinal cord injury, aiming at laying the experimental foundation for the clinical exploration of new paths for the treatment of spinal cord injury.

## 2 MATERIALS AND METHODS

### 2.1 Laboratory animals

Thirty adult Wistar rats, male and female, with body mass of 210‐240 g, were selected as experimental animals, which were provided Shanghai Slake Co. The experimental group was divided into control group and experimental group, 15 rats each. The experimental group was treated with spinal cord injury and localized transplantation of silver nanohydrogel nerve conduits containing cBMMSC, while the control group was treated with spinal cord injury and localized transplantation of silver nanohydrogel nerve conduits not containing cBMMSC. Both groups were anaesthetised 4 weeks after transplantation and executed by open‐chest 4% paraformaldehyde perfusion. The spinal cord tissues were serially frozen transverse sectioned at a thickness of 5 μm, fixed in cold acetone, hydrogen peroxide was used to remove endogenous peroxidase, and serum was closed. The study was approved and adopted by the Animal Ethics Committee (Approval No.20240625143519505043).

### 2.2 Treatment methods

#### 2.2.1 Preparation of cBMMSC

In this study, cBMMSC, which we previously obtained and isolated during surgery, were selected and placed in culture medium containing foetal bovine serum, glutamine phthalamide, penicillin, and concanavalin streptomycin, and cultured in an incubator containing 5% CO_2_ and 37°C saturated humidity. After the 1st passaging, the cells were passaged every 3 days at a ratio of 1:3 for expansion culture. Unapproximated cells were discarded, cells were passaged for 3 to 6 generations, and cells were washed 3 times during transplantation, and the final concentration of 1 × 105/μL was adjusted and set aside.

#### 2.2.2 Spinal cord injury modelling

The rats were fasted with water the night before surgery, and 1% pentobarbital (40 mg/kg) was injected intraperitoneally on the day of surgery. After successful anaesthesia, the rats were fixed on a hot cotton padded rat board and the limbs were spread out in a relaxed position. To determine the surgical segment (the most obvious spine between the two scapulae in the prone position of the rat is the T_2_ spine, and this is the marking point to the lower thoracic segment to touch the spines in turn and count, when the T_9_ spine is touched the spine protrudes backward and becomes significantly smaller, and the next spine is the T_10_ spine), the mouse hair of the surgical area was shaved with care (operated gently, to prevent damage to the skin), and after disinfecting and spreading the towel, the spine was made a longitudinal movement centering on T_9‐10_ with the aid of the head mounted magnifying glass, and a longitudinal movement of the spine was made with T_9‐10_ as the center along the spinal midline. T_10_ as the center, make a longitudinal incision of about 2.5 cm in length, remove the fat, and separate the paravertebral muscles along both sides of the spine to expose the T_8‐11_ spinous processes. Using a skin spreader, the skin and muscles were spread open, and the soft tissues such as muscles remaining on the vertebral plate were cleaned with a surgical blade to fully expose the field of view. Flex the dorsum of the mouse and use blood. The T_9_ spine was gently lifted up with tube forceps, so that the intervertebral plate space of T_10_/T_11_ was opened as much as possible, and the T_10_ spine and vertebral plate were removed by biting with pointed micro biting forceps and enlarged to both sides and the T_9_ region to fully expose the T_9‐10_ spinal cord. The dura mater was opened with microscopic forceps and microsurgical shears, and the spinal cord was gently passed ventrally through the spinal cord with a blunt neural hook (homemade), which gently hooked up the spinal cord and detached all the connecting tissues, and a section of the spinal cord 3 mm long was carefully resected. A small gelatin sponge was used to gently stop the bleeding and a razor blade was used to scrape away any possible residual neural tissue from the resected segment to confirm complete transection of the spinal cord.^7^

#### 2.2.3 Nanosilver hydrogel nerve conduit cBMMSC transplantation

Nanosilver hydrogel nerve catheters (purchased from Shanghai Research Biotechnology Co., Ltd.) were prepared in advance, and the catheters were sterilized before transplantation and cut into small catheters with a length of 3 mm and a diameter of 3 mm (containing 50 catheters with a diameter of 100 μm). In vivo transplanted cells were planted in the catheter in the same way as in vitro cell implantation, and each catheter received 10 μL of cell suspension containing 1 × 10^5^ cells. In the experimental group, nerve catheters implanted with cBMMSC were placed between the two severed ends of the spinal cord injury to make a tight connection with the spinal cord, whereas the control group was not given the catheters or cell transplantation. After seeing no obvious active bleeding, the dura, muscle and skin were sutured sequentially and the incision was closed. After surgery, the rats were resuscitated on hot cotton pads until they became active and were fed and watered.

### 2.3 Detection Methods

#### 2.3.1 MAB1281 staining with NSE labeling

NSE expression in MAB1281‐positive cells was detected by immunodouble staining. Sections were first treated with MAB1281 monoclonal antibody, added anti‐NSE (1:80 dilution) monoclonal antibody at room temperature for 2 h, then incubated with biotin‐labelled goat anti‐mouse for 30 min at room temperature, rinsed with PBS, and then incubated with horseradish peroxidase‐labelled streptavidin working solution for 30 min at room temperature, and then developed with AEC, and then observed by light microscope. The expression of NSE on the MAB1281‐positive cells was in the form of brown or brownish‐yellow. Brown or brownish yellow.

#### 2.3.2 Neuronal and axonal regeneration assays

Paraffin sections were dewaxed and hydrated with gradient hydration. 0.01 mol/L PBS washed 3 times, each time for 2 min, and 0.3% by volume TritonX‐100 solution was added for 30 min at room temperature. 0.01 mol/L PBS washed 3 times, each time for 2 min. Normal blocked serum was added, and the serum was incubated for 30 min at room temperature. the liquid was aspirated, and the primary antibody of rat anti‐mouse BrdU was added (1:2500) overnight at 4°C. 0.01 mol/L PBS was washed 3 times, each time for 2 min, and industrial anti‐TRITC labelled sheep anti‐mouse IgG was added. Add normal closed serum, 30 min at room temperature, aspirate the liquid, drop rat anti‐mouse BrdU primary antibody (1:2500) overnight at 4°C. Wash with 0.01 mol/L PBS 3 times, 2 min each time, add worker anti‐TRITC labelled goat anti‐mouse IgG, 1 ~ 2 h at 37°C, aspirate the secondary antibody, wash with 0.01 mol/L PBS 3 times, 2 min each time, and add 0.3% by volume TritonX‐100 solution for 30 min at room temperature. 0.01 mol/L PBS wash 3 times, 2 min each time, add 0.3% by volume TritonX‐100 solution for 30 min at room temperature. PBS was washed 3 times, each time for 2 min, and normal closed serum was added, and scanned under a laser confocal microscope (Leica, STELLARIS, Germany). One hundred spinal cord sections were taken from each group, and the number of BrdU‐positive cells and the length of axons on each section were recorded.

#### 2.3.3 HE staining

The spinal cord tissue samples were fixed in paraformaldehyde solution and made into tissue specimens, which were dehydrated by gradient alcohol, put into xylene transparent, dipped in wax, and embedded buccolingually. Wax blocks were serially sectioned at a thickness of 3 μm. conventional xylene, gradient alcohol dewaxing to water, HE staining with eosin staining solution, and neutral gum sealing. The cavities and scar formation were observed under light microscope.

#### 2.3.4 immunofluorescence assay

Spinal cord sections were oven‐dried at 60°C for 30 min, dehydrated sequentially with gradient alcohol, rinsed 3 times with PBS for 5 min each time; 3% hydrogen peroxide methanol was used to eliminate the endogenous peroxidase activity, incubated at room temperature for 20 min; rinsed 3 times with PBS for 5 min each time; 5% goat serum was used to block the non‐specific staining induced by the endogenous biotin, incubated at room temperature for 30 min; primary antibody (Nestin, NSE, NF200, GFAP) serum was added dropwise and incubated at 37°C for 60 min; PBS was rinsed three times, each time for 5 min; DAB colour development was controlled under a microscope for 1 ~ 5 min: rinsed with tap water, and the results were observed under a light microscope, and the OD value of the immunofluorescent‐stained cells was detected by using the accompanying LEICA QWin Image Processing and Analysis System for image analysis. The OD value of the immunofluorescence staining positive cells was detected. When measuring, 3 measurement points were taken for each cell, and the amount of pixels measured at each measurement point was the same, and 5 positive cells were measured at the corresponding sites in each section. The OD value of the immunofluorescence‐stained neural cells in the spinal cord was determined by subtracting the average of the measured values from the measured values of the corresponding negative control slices and taking the opposite of that value as the final OD value of the immunofluorescence‐stained neural cells in the spinal cord.

#### 2.3.5 Western blot sensing

The motor fibre neurotransmitter acetylcholine generation‐limiting enzyme (ChAT) and the sensory fibre neurotransmitter glutamate synthase (GOGAT) were first extracted from the spinal cord tissue samples using special protein extraction reagents; the extracted protein samples were subjected to SDS‐PAGE gel electrophoresis to separate the proteins according to molecular weights; the proteins were transferred from the gel to polypropylene or nitrocellulose membranes The proteins are transferred from the gel to a polypropylene or nitrocellulose membrane, and then proteins such as bovine serum albumin (BSA) are used to block the non‐specific binding sites and reduce false‐positive results; a specific primary antibody is added to allow for specific binding to the target protein; unbound primary antibodies are washed away to reduce non‐specific binding; a secondary antibody labelled with a fluorescein or an enzyme is added to bind to the primary antibody and form a complex; the unbound secondary antibody is washed away to reduce the background signal; and the fluorescein or enzyme is detected to observe the signal of the proteins. The added fluorescein or enzyme is detected to observe the protein signals and analyse the presence of ChAT and GOGAT proteins and their expression levels.

#### 2.3.6 BBB score

BBB motor function scores were performed on both groups at 1, 2, 3 and 4 weeks post‐transplantation on a scale of 0–21, with a score of 0 indicating no visually visible hindlimb movement changes, and a score of 21 indicating a sustained plantar gait and sustained coordination with forelimb movement changes; sustained grip of toes during forward movement; a predominantly parallel to the body transplantation posture when the foot is in contact with the ground or when the palm is lifted; sustained lifting of the tail, and torso Sustained stabilization. Higher scores suggest better recovery of motor function.^8^

#### 2.3.7 Somatosensory evoked potential (SEP) and motor evoked potential (MEP) detection

After anaesthetised with 10% chloral hydrate and fixed the limbs, the MEP and SEP were detected. SEP detection: the electrode was placed at about 0.5 cm posterior to the sensory area of the hind limb cortex under the scalp, and current stimulation (DC square wave) was given at a frequency of 3 Hz, wave width of 0.2 ms, intensity of 5–15 mA and superimposed number of times of 50–60 times. MEP detection: the electrode was placed at the motor area of the cortex, intensity of 40 mA, wave width of 0.1 ms, frequency of 1 Hz, scanning speed of 5 ms/D and superimposed number of times of 300–500 times. MEP was detected by placing an electrode on the motor area of the cerebral cortex with an intensity of 40 mA, wave width of 0.1 ms, frequency of 1 Hz, scanning speed of 5 ms/D, superimposed number of times of 300–500, and sensitivity of 5 μV/D. The amplitude and the latency of corresponding evoked potentials were recorded in detail for the two groups after the current stimulation.

### 2.4 Statistical methods

Data were analysed using the statistical analysis software SPSS 17.0, and measurements were expressed as arithmetic mean ± standard deviation and statistically tested using one‐way ANOVA. Each group of variables contained at least three valid values, and the significant difference level was set at *p* < 0.05 to indicate that the data were statistically significantly different.

## 3 RESULTS

### 3.1 Immunohistochemical Results

#### 3.1.1 Differentiation of cBMMSC to neuron‐like cells in nerve conduits after transplantation

**FIGURE 1:**
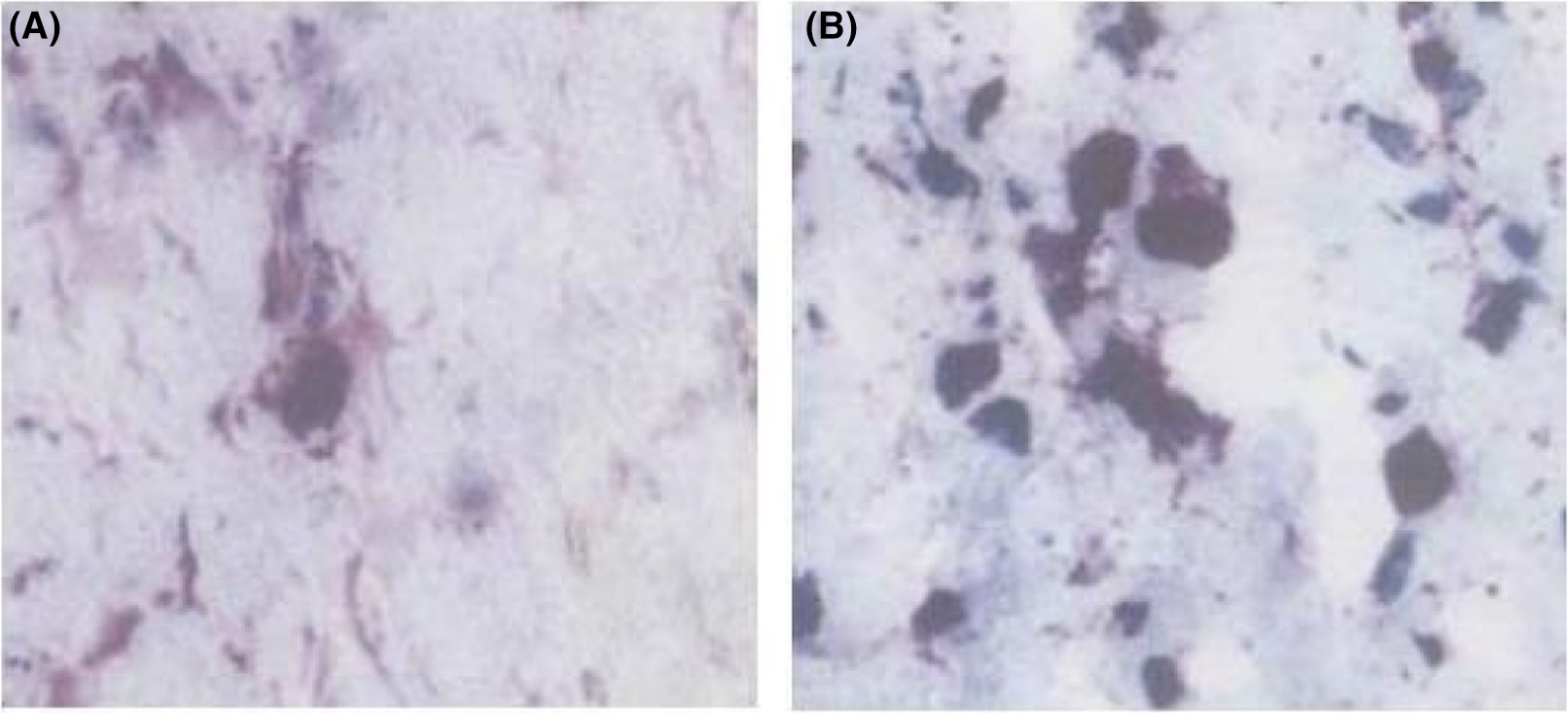
NSE expression of MAB1281‐positive cells after 4 weeks of transplantation in the (A) control group and (B) in the experimental group. Scale bar: 50 μm.

Immunohistochemical staining results showed that 4 weeks after transplantation, MAB1281‐positive cells were seen to survive in both groups, and MAB1281‐positive cells were aggregated around the spinal cord injury tissues. The sepia component in the MAB1281 positive cells of the experimental group was darker and more abundant than that of the control group, suggesting that their NSE content was more abundant than that of the control group. See Figure 1.

#### 3.1.2 Regeneration of neurons and axons localized to spinal cord injury in two groups after transplantation

**FIGURE 2:**
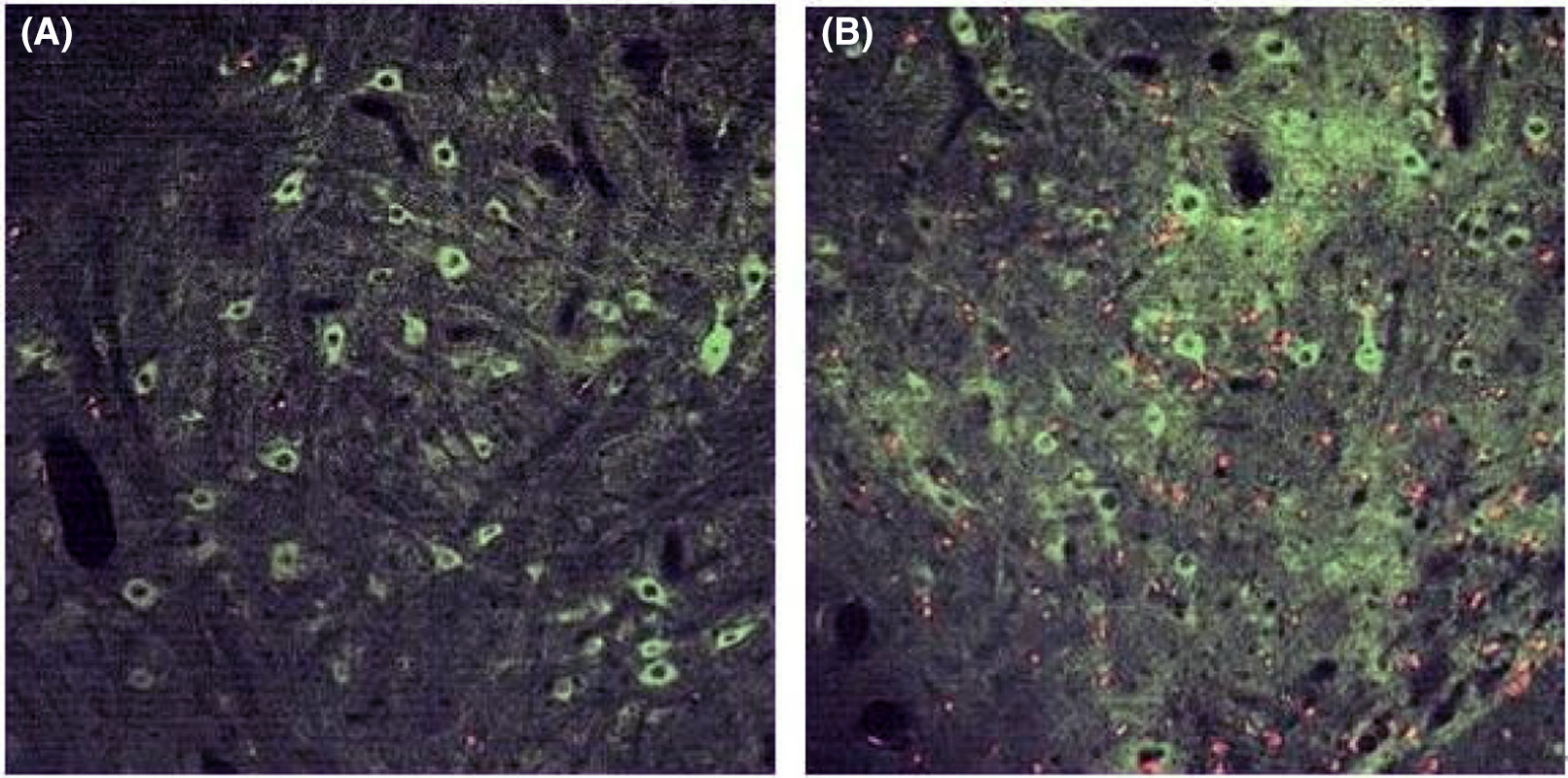
Expression of BrdU‐positive cells after 4 weeks of transplantation (A) in the control group and (B) in the experimental group. Scale bar: 50 μm.

**FIGURE 3:**
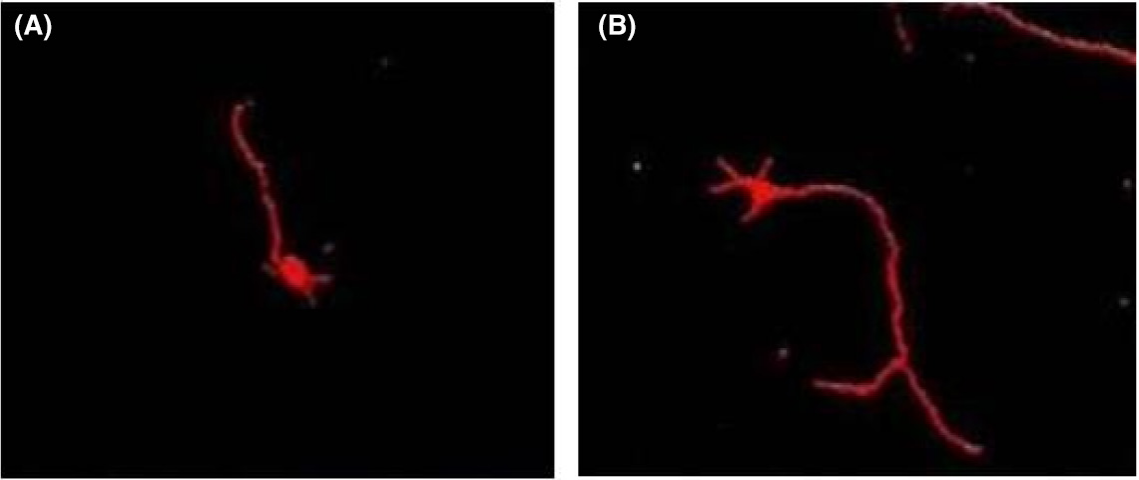
Axon length of (A) control neurons and (B) experimental neurons after 4 weeks of transplantation. Scale bar: 10 μm.

Immunofluorescence staining and laser confocal photography showed that 4 weeks after transplantation, only a very few BrdU‐positive cells were visible in the normal control group (red‐dotted portion of Figure 2), which were distributed in the white and grey matter; more BrdU‐positive cells were visible in the experimental group, which were distributed in the white and grey matter; the number of BrdU‐positive cells in the experimental group was significantly more than that of the control group [(70.70 ± 11.49) vs. (2.73 ± 1.46), *p* < 0.05], difference in means about 67.97, and the length of the axon (the red line portion of Figure 3) was significantly longer than the control group [(205.08 ± 18.57) vs. (101.19 ± 10.43), *p* < 0.05], difference in means about 103.89. See Figures 2, 3 and Table 1.

**TABLE 1:** Comparison of the number of BrdU‐positive cells and neuronal axon length between the two groups 4 weeks after transplantation(±s).

Groups	*n*	Number of BrdU‐positive cells (individuals)	Neuronal axon length(μm)
Control group	15	2.73 ± 1.46	101.19 ± 10.43
Experimental group	15	70.70 ± 11.49	205.08 ± 18.57
*t*		22.728	18.891
*p*		0.000	0.000

#### 3.1.3 Comparison of neuron‐like marker expression levels between the two groups after transplantation

**FIGURE 4:**
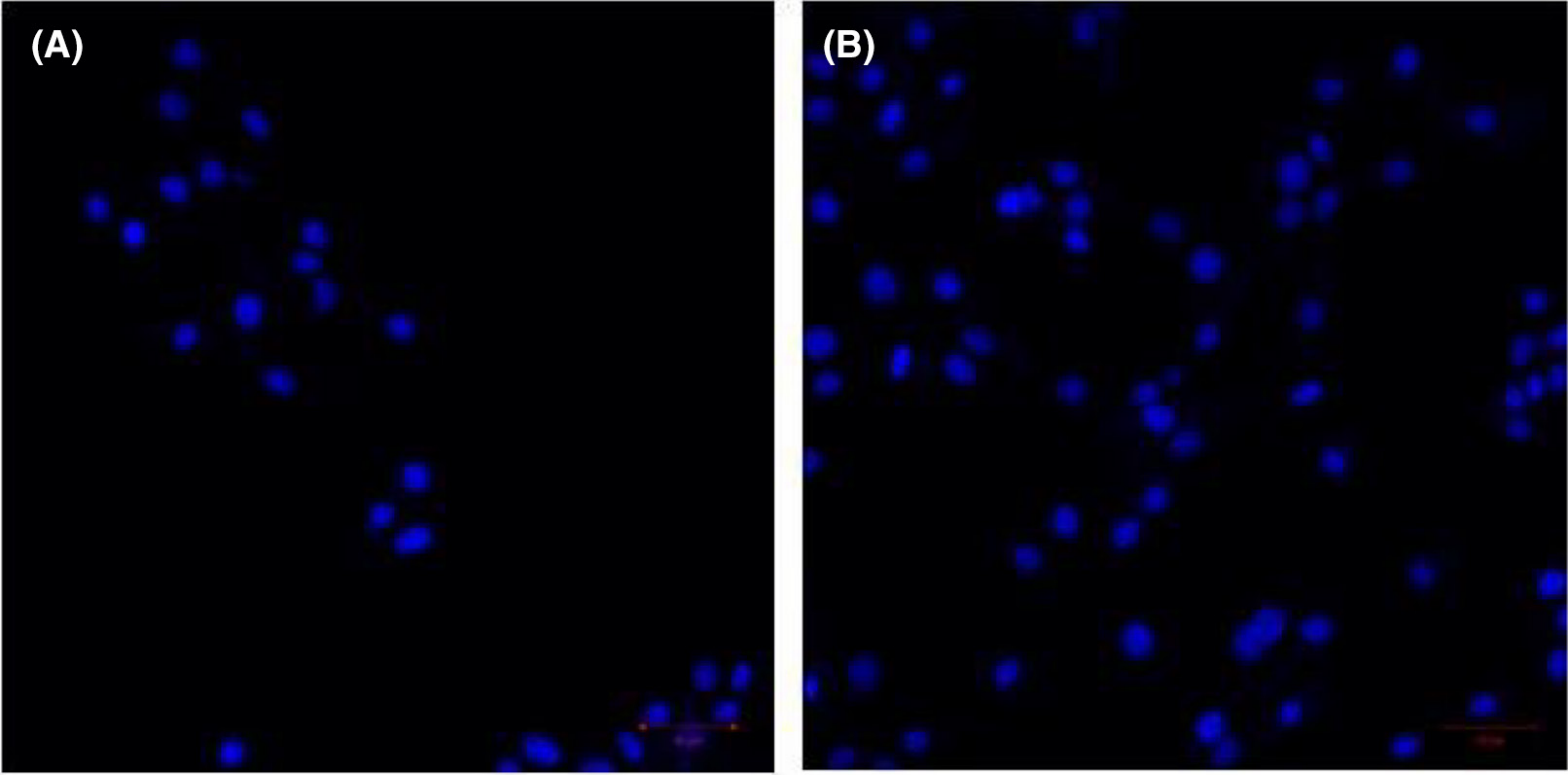
Expression of Nestin‐positive cells after 4 weeks of transplantation (A) in the control group and (B) in the experimental group. Scale bar: 50 μm.

**FIGURE 5:**
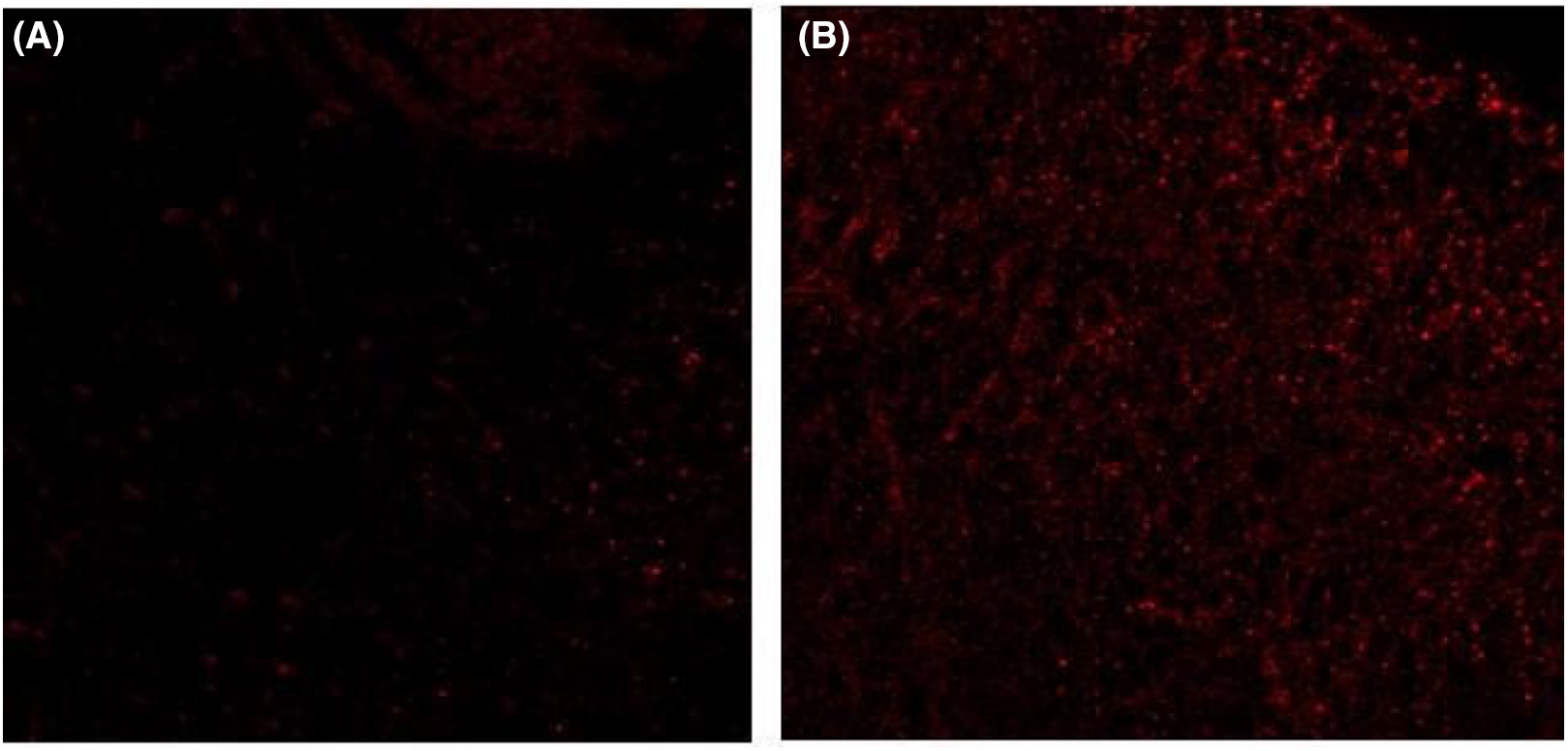
Expression of NSE‐positive cells after 4 weeks of transplantation (A) in the control group and (B) in the experimental group. Scale bar: 50 μm.

**FIGURE 6:**
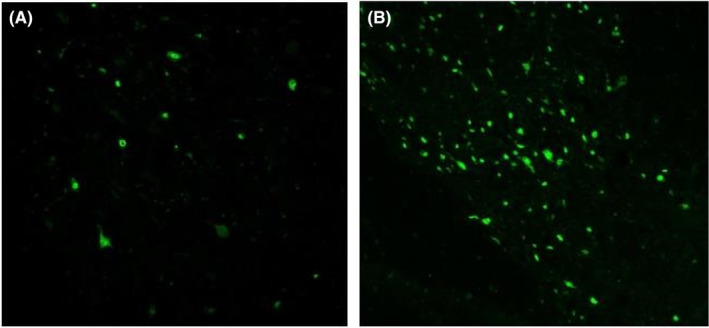
Expression of NF200‐positive cells after 4 weeks of transplantation (A) in the control group and (B) in the experimental group. Scale bar: 50 μm.

**FIGURE 7:**
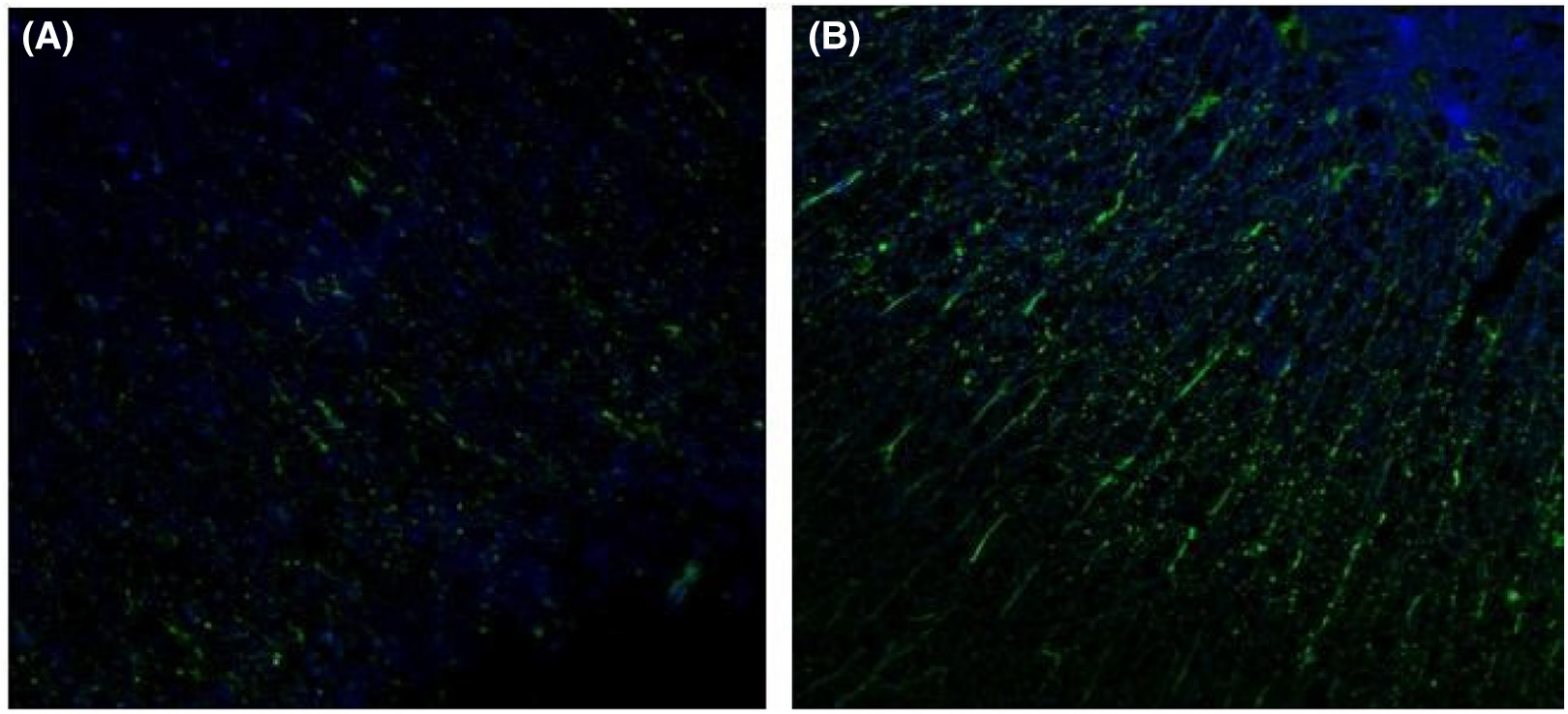
Expression of GFAP‐positive cells after 4 weeks of transplantation (A) in the control group and (B) in the experimental group. Scale bar: 50 μm.

Immunofluorescence staining results showed that 4 weeks after transplantation, a large number of Nestin‐positive cells existed around the spinal cord ventricular membranes in the experimental group, with cell protrusions showing and strong Nestin staining, whereas there were few in the control group, and the OD value of Nestin‐positive cells in the experimental group was significantly higher than that of the control group [(5.14 ± 0.31) vs. (2.65 ± 0.13), *p* < 0.05], difference in means about 2.49; the NSE‐positive expression area was concentrated in the grey matter of the spinal cord, and the expression was significantly reduced in the white matter; the application of goat With the application of goat anti‐rabbit‐FITC antibody, the stem cells showed obvious bright red fluorescence and the expression result was significant; the OD value of NSE‐positive cells in the experimental group was significantly higher than that of the control group [(6.42 ± 0.50) vs. (2.55 ± 0.27), *p*<0.05], difference in means about 3.87. NF200 was only expressed in the axons of neurons in the two groups, and the stem cells showed obvious yellow‐green fluorescence under immunofluorescence staining, and the expression result was significant; the OD value of NF200‐positive cells in the experimental group was significantly higher than that of the control group [(7.58 ± 1.56) vs. (2.46 ± 0.10), *p*<0.05], difference in means about 5.12. GFAP was only expressed in the axons of the two groups. GFAP positive areas were concentrated in the grey matter of the spinal cord, with less expression in the white matter; applying goat anti‐rabbit‐FITC antibody, the stem cells showed obvious blue fluorescence, with significant expression results; dropping goat anti‐mouse‐Cy3 antibody on the stem cells showed obvious yellow‐green fluorescence, with significant expression results. The OD value of GFAP positive cells in the experimental group was significantly higher than that of the control group [(6.18 ± 0.56) vs. (2.72 ± 0.15), *p*<0.05], difference in means about 3.46. See Figures 4, 5, 6, 7 and Table 2.

**TABLE 2:** Comparison of OD values of Nestin, NSE, NF200 and GFAP‐positive cells in two groups after transplantation(±s).

Groups	*n*	Nestin	NSE	NF200	GFAP
Control group	15	2.65 ± 0.13	2.55 ± 0.27	2.46 ± 0.10	2.72 ± 0.15
Experimental group	15	5.14 ± 0.31	6.42 ± 0.50	7.58 ± 1.56	6.18 ± 0.56
*t*		28.688	26.377	12.685	23.115
*p*		0.000	0.000	0.000	0.000

**FIGURE 8:**
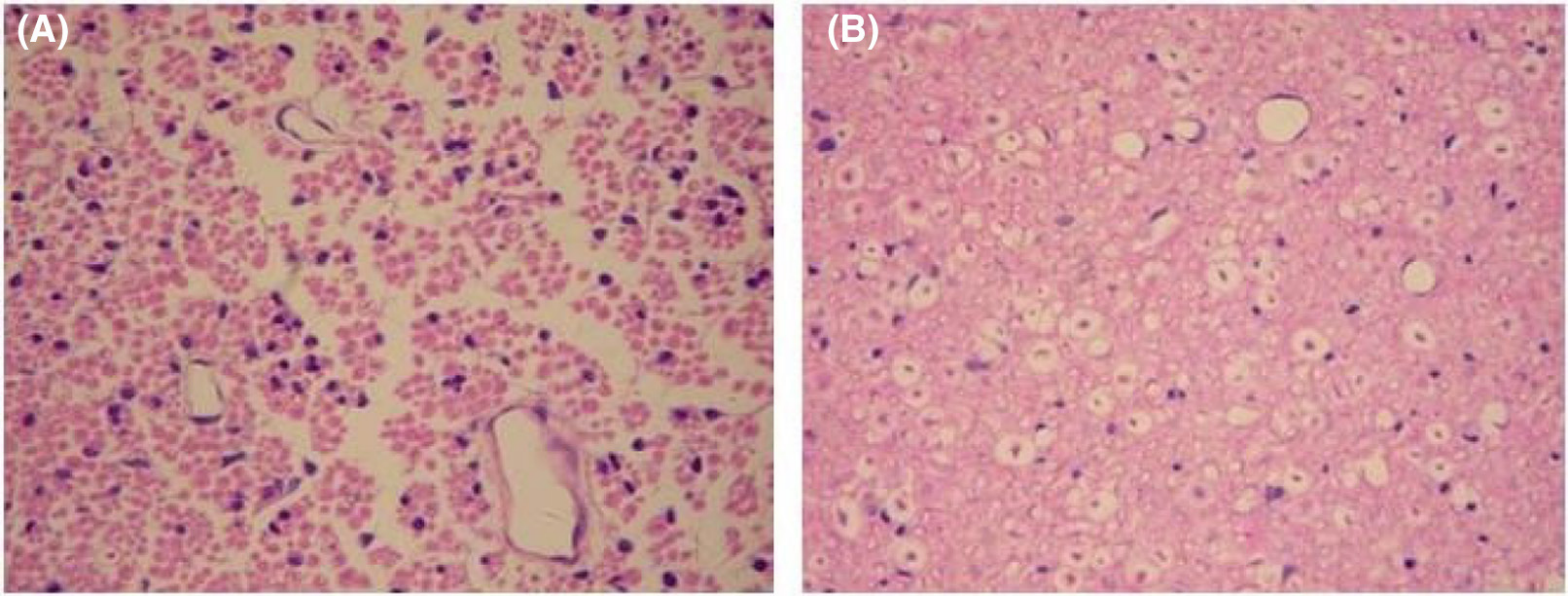
Changes in HE staining of spinal cord sections after 4 weeks of transplantation (A) in the control group and (B) in the experimental group. Scale bar: 50 μm.

### 3.2 Cell proliferation and regeneration results

HE staining results showed that 4 weeks after transplantation, the control group had slight enlargement of neuronal cells and glial cells, widening of the perivascular gap, reduction of neuronal cells, degeneration and necrosis of a large number of nerve fibres, and sparsely reticulated changes in the white matter. Inflammatory cell infiltration, glial cell proliferation and scar formation in the injury area. In the experimental group, necrosis of nerve fibres was also seen, blank formation in some areas, demyelinating changes in axons, which were significantly reduced compared to the control group, and a large number of glial cells and nerve fibres were seen in the white matter. In the control group, the voids formed after nerve necrosis could also be seen, which were more numerous and larger than those in the experimental group. See Figure 8.

### 3.3 Immunoblotting results

The results of Western blot detection showed that 4 weeks after transplantation, the expression levels of ChAT and GOGAT in the spinal cord slices of the experimental group were significantly higher than those of the control group [(14.52 ± 4.79) vs. (7.22 ± 2.42); (30.54 ± 6.03) vs. (14.68 ± 5.98), *p*<0.05], difference in means about 7.30, 15.86. See Table 3.

**TABLE 3:** Comparison of ChAT and GOGAT expression levels in the two groups after transplantation in both groups(±s).

Groups	*n*	ChAT(μg/mL)	GOGAT(U/L)
Control group	15	7.22 ± 2.42	14.68 ± 5.98
Experimental group	15	14.52 ± 4.79	30.54 ± 6.03
*t*		5.268	7.233
*p*		0.000	0.000

### 3.4 Neurofunctional recovery assessment

#### 3.4.1 Comparison of BBB scores between the two groups after transplantation

After 1, 2, 3 and 4 weeks of transplantation, intra‐group comparison: there was no significant change in the BBB score of the control group, while the BBB score of the experimental group gradually increased and reached the peak after 4 weeks, with a significant difference (*p* < 0.05). Comparison between groups: the BBB scores of the experimental group were significantly higher than those of the control group at all time points (*p* < 0.05). See Table 4.

**TABLE 4:** Comparison of BBB scores between the two groups after transplantation (point, ±s).

Groups	*n*	1week	2week	3week	4week	*F*	*p*
Control group	15	3.36 ± 1.42	3.69 ± 1.05	3.25 ± 1.36	3.66 ± 1.82	0.542	0.854
Experimental group	15	6.61 ± 2.32	8.88 ± 2.47	12.68 ± 3.25	16.11 ± 3.72	9.865	0.003
*t*		4.628	7.489	10.367	11.643		
*p*		0.000	0.000	0.000	0.000		

#### 3.4.2 Comparison of SEP and MEP between the two groups after transplantation

After 4 weeks of transplantation, the SEP and MEP latency of the experimental group was significantly shorter [(14.83 ± 1.19) vs. (38.47 ± 2.88), *p*<0.05], difference in means about 23.64, and the wave amplitude was significantly larger than that of the control group [(4.64 ± 0.14) vs. (1.96 ± 0.17), *p*<0.05], difference in means about 2.68. See Table 5.

**TABLE 5:** Comparison of SEP and MEP between the two groups after transplantation(±s).

Groups	*n*	Incubation period(ms)	Amplitude(μV)
Control group	15	38.47 ± 2.88	1.96 ± 0.17
Experimental group	15	14.83 ± 1.19	4.64 ± 0.14
*t*		29.381	47.131
*p*		0.000	0.000

## 4 DISCUSSION

The external causes of spinal cord injury often include spinal dislocation and fracture, and the internal causes include infection, tumour compression, etc. The main pathological changes of spinal cord injury include neuronal necrosis, degeneration of nerve axons and demyelination changes, which are able to impede the conduction of nerve signals.^9^ Therefore, the most critical aspect of treating spinal cord injury is to protect the nerves and promote nerve regeneration. The traditional concept is that the central nervous system cannot be regenerated and repaired after injury, but some studies have confirmed that the microenvironment of the central nervous system can inhibit the repair of injured nerves, retard the growth of neurons in the spinal cord, and that the lack of nerve regeneration factors and the increased secretion of nerve regeneration inhibitory factors lead to the formation of physical barriers in the spinal cord, such as cavities, cysts and scars.^10^, ^11^ People have successively used medication, surgery, physical therapy, nerve transplantation and other means to treat spinal cord injury, but these treatments have failed to achieve satisfactory therapeutic effects. Stem cells are cells with strong self‐renewal ability and potential to differentiate into multiple functional cells. Human cBMMSC are bone marrow stromal stem cells, a subpopulation of cells found in the bone marrow stroma of mammals with multiple differentiation potentials to form bone, cartilage, adipose, neural and myoblasts, which have a fast multiplication rate and good differentiation potentials.^12^ It has been demonstrated that cBMMSC can differentiate into various adult cells after induced culture. It has successfully induced differentiation toward neuronal cells and can secrete neurotrophic factors in vitro.^13^ In the present study, a novel nanosilver hydrogel nerve conduit material was used as a medium, into which cBMMSC were injected and transplanted in a rat model of spinal cord injury to observe the effect of spinal cord repair and the results were as follows.

In recent years, there are increasing reports that cBMMSC can survive in injured neural tissues and differentiate into neuron‐like and astrocyte‐like cells to promote the recovery of neurological functions, showing good prospects for application. zhang H et al.^14^ found that cBMMSC could differentiate into neuron‐like and neuroglial cells after being injected into the lateral ventricle of neonatal rats, which opens the possibility of utilizing cBMMSC for the treatment of several neurological diseases, such as progeria dementia and Parkinson's disease, among other neurological disorders, offers the possibility of utilizing cBMMSC for the treatment of Alzheimer's disease and Parkinson's disease. However, their application is severely restricted due to their limited sources, difficulties in obtaining materials, or promotion of legal and ethical issues. In this study, we found that MAB1281‐positive cells were NSE‐positive, confirming that surviving cBMMSC can differentiate to neuron‐like and astrocyte‐like cells, which is consistent with the findings of the above studies. BrdU is 5 a bromodeoxyuridine, and pyrimidine is the basic component of DNA, integrated in the DNA of cells during the formation of newborn cells, so BrdU nucleus‐positive cells are newborn neuronal cells,^15^ this study found that BrdU‐positive cells of the experimental group after transplantation of cBMMSC were significantly more than those of the control group, and the length of axon was significantly longer than that of the control group, which suggests that cBMMSC could promote the neuron and axon regeneration, which may be related to the changes in the microenvironment of neuronal cells in the spinal cord injury area after transplantation of cBMMSC based on nanosilver hydrogel nerve conduit. nanosilver hydrogel material is biocompatible, which can significantly reduce the release of haemorrhage, necrosis and inflammatory mediators in the spinal cord injury area, and provide a good local endo‐environment for the differentiation of cBMMSC into newborn neuronal cells.^16^ In addition, this paper found that 4 weeks after transplantation, the expression levels of Nestin, NSE, NF200, GFAP, ChAT, GOGAT and BBB score of the experimental group were significantly higher than those of the control group; the scarring and cavitation of the spinal cord slices were significantly lighter than those of the control group; the latency time of SEP and MEP was significantly shorter than that of the control group, and the amplitude of the waveforms was significantly greater than that of the control group, and the above results suggest that cBMMSC transplanted based on nano‐silver These results suggest that cBMMSC transplantation based on nanosilver hydrogel nerve conduit is effective in repairing spinal cord injury. The results may be related to the protective and regenerative effects of cBMMSC on nerves, and promote the recovery of neuromotor and sensory functions. cBMMSC transplantation of the injured spinal cord replaces the functional role of the original nerve cells, and promotes the recovery of nerve function, while a small number of them differentiate in the direction of the neural lineage cells to replace apoptotic, necrotic neurons and promotes the myelination of the axons.^17^ In addition, cBMMSC have the effect of inhibiting the activation of microglia and attenuating the immune‐inflammatory response after nerve injury. After transplantation, cBMMSC may have the function of secreting cytokines and neurotrophic factors, including GDNF, NT‐3, IL‐6, BDNF, bFGF, FGF20, neutrophil activating protein‐2 and glucocorticoid‐induced tumour necrosis factor‐3, etc., which directly produces a neuroprotective effect.^18^ It also promotes local angiogenesis to form the reconstruction and recirculation of circulating blood pathways in the injured spinal cord, while secreting vascular endothelial cell growth factor receptor‐3 on the one hand, and possibly differentiating into endothelial cells on the other.^19^ In addition, the cBMMSC transplantation section based on nanosilver hydrogel nerve conduit plays the role of neuronal replacement and bridging in the specific environment after spinal cord injury, which can reduce the barrier obstruction effect of glial scarring physically and chemically, and rebuild the neural circuits^20^; the cBMMSC differentiate to oligodendrocytes, encircle the axons and form myelin sheaths, and the secreted related growth factors and trophic factors actively participate in the inter‐synaptic neural connections, thus restoring the integrity of neural electrical conduction.^21^ Zhou Y et al.^22^ found that cBMMSC can rapidly expand in vitro, survive and differentiate into neuronal cells at the site of spinal cord injury in rats after transplantation, which can effectively promote the recovery of motor function and regeneration of neuronal axons after spinal cord injury. tian M et al.^23^ on the biological properties and ultrastructure of cBMMSC found that the cell morphology of cBMMSC is similar to that of bone marrow MSCs, and the majority of the morphology is similar to fibroblasts. MSCs, the vast majority of the morphology similar to fibroblasts, the stable growth of passaged cells, active cell proliferation and strong self‐renewal ability; microscopy confirms that there are abundant organelles, a large proportion of nucleoplasm and multiple nucleoli are visible, which indicates that the cells are metabolically active and have a low degree of differentiation and microvilli are seen on the surface of the cells, which can increase the adsorption and absorption ability of the cells; ELISA assay confirms that cBMMSC are able to ELISA test confirmed that cBMMSC could secrete cytokines such as VEGF, IGF‐1 and HGF. The above conclusions are consistent with the results of the present study. In addition, the sample size of this study is small and the research object is single, the specific molecular mechanism of the repair of spinal cord injury by nanosilver hydrogel nerve conduit and cBMMSC needs to be confirmed by more in‐depth and better multi‐center and large‐sample studies.

In conclusion, cBMMSC transplantation based on nanosilver hydrogel nerve conduit was effective in repairing spinal cord injury, promoting neuronal and axonal regeneration and restoring neuromotor and electrophysiological functions.

## AUTHOR CONTRIBUTIONS

**Zhuxiao Tang:** Conceptualization (equal); data curation (equal); formal analysis (equal); investigation (equal); methodology (equal); software (equal); supervision (equal); validation (equal); visualization (equal); writing – original draft (equal); writing – review and editing (equal). **Yahui Ye:** Conceptualization (equal); data curation (equal); formal analysis (equal); methodology (equal); software (equal); supervision (equal); validation (equal); visualization (equal); writing – original draft (equal); writing – review and editing (equal). **Kaichuang Yang:** Data curation (equal); formal analysis (equal); investigation (equal); methodology (equal); validation (equal); visualization (equal); writing – review and editing (equal). **Xi Guo:** Data curation (equal); formal analysis (equal); investigation (equal); methodology (equal); supervision (equal); validation (equal); visualization (equal); writing – review and editing (equal). **Xin Gao:** Data curation (equal); formal analysis (equal); investigation (equal); validation (equal); visualization (equal); writing – review and editing (equal). **Cheng Wu:** Data curation (equal); formal analysis (equal); investigation (equal); software (equal); validation (equal); visualization (equal); writing – review and editing (equal). **Jingyu Wang:** Data curation (equal); formal analysis (equal); investigation (equal); validation (equal); visualization (equal); writing – review and editing (equal). **Deqing Peng:** Formal analysis (equal); funding acquisition (equal); project administration (equal); supervision (equal); validation (equal); visualization (equal); writing – review and editing (equal).

## FUNDING INFORMATION

This study was funded by the Natural Science Foundation of Zhejiang Province (LQ21H090016), the Scientific Research Fund of Zhejiang Provincial Education Department (Y202146121) and the General Project Funds from the Health Department of Zhejiang Province (2023KY002).

## CONFLICT OF INTEREST STATEMENT

The authors declare no conflict of interest.

## CONSENT FOR PUBLISH

All of the authors have consented to publish this research.

## Data Availability

The data are free access to available upon request.
